# Supplementary material for: Pyruvate Dehydrogenase Kinase 1 inhibition mediated oxidative phosphorylation enhancement in cartilage promotes osteoarthritis progression
Source: BMC Musculoskelet Disord. 2023 Jul 20;24:597. doi: 10.1186/s12891-023-06585-6 (PMC10357736; doi:10.1186/s12891-023-06585-6)
Supplement: Supplementary file 1 — Additional file 1. [file 12891_2023_6585_MOESM1_ESM.zip › MF.Gsea.1653627316242/GOMF_ATP_HYDROLYSIS_ACTIVITY.html]

Details for gene set GOMF\_ATP\_HYDROLYSIS\_ACTIVITY[GSEA]

|  || Dataset | 55457\_collapsed\_to\_symbols.cls.cls #OA\_versus\_normal.cls.cls #OA\_versus\_normal\_repos |
| Phenotype | cls.cls#OA\_versus\_normal\_repos |
| Upregulated in class | normal |
| GeneSet | GOMF\_ATP\_HYDROLYSIS\_ACTIVITY |
| Enrichment Score (ES) | -0.3108416 |
| Normalized Enrichment Score (NES) | -1.5387743 |
| Nominal p-value | 0.0013717421 |
| FDR q-value | 0.16336924 |
| FWER p-Value | 1.0 |
Table: GSEA Results Summary

  

Fig 1: Enrichment plot: GOMF\_ATP\_HYDROLYSIS\_ACTIVITY      
 Profile of the Running ES Score & Positions of GeneSet Members on the Rank Ordered List

  

| SYMBOL | TITLE | RANK IN GENE LIST | RANK METRIC SCORE | RUNNING ES | CORE ENRICHMENT || 1 | NVL | nuclear VCP like [Source:HGNC Symbol;Acc:HGNC:8070] | 73 | 0.956 | 0.0054 | No |
| 2 | NLRP1 | NLR family pyrin domain containing 1 [Source:HGNC Symbol;Acc:HGNC:14374] | 200 | 0.819 | 0.0049 | No |
| 3 | MCM5 | minichromosome maintenance complex component 5 [Source:HGNC Symbol;Acc:HGNC:6948] | 351 | 0.724 | 0.0015 | No |
| 4 | MLH1 | mutL homolog 1 [Source:HGNC Symbol;Acc:HGNC:7127] | 497 | 0.660 | -0.0023 | No |
| 5 | ATP6V1G1 | ATPase H+ transporting V1 subunit G1 [Source:HGNC Symbol;Acc:HGNC:864] | 515 | 0.655 | 0.0040 | No |
| 6 | PSMC2 | "proteasome 26S subunit, ATPase 2 [Source:HGNC Symbol;Acc:HGNC:9548]" | 559 | 0.638 | 0.0080 | No |
| 7 | PFN2 | profilin 2 [Source:HGNC Symbol;Acc:HGNC:8882] | 560 | 0.638 | 0.0155 | No |
| 8 | VWA8 | von Willebrand factor A domain containing 8 [Source:HGNC Symbol;Acc:HGNC:29071] | 568 | 0.633 | 0.0223 | No |
| 9 | HSPA2 | heat shock protein family A (Hsp70) member 2 [Source:HGNC Symbol;Acc:HGNC:5235] | 662 | 0.603 | 0.0219 | No |
| 10 | MSH2 | mutS homolog 2 [Source:HGNC Symbol;Acc:HGNC:7325] | 739 | 0.584 | 0.0227 | No |
| 11 | MCM2 | minichromosome maintenance complex component 2 [Source:HGNC Symbol;Acc:HGNC:6944] | 888 | 0.546 | 0.0174 | No |
| 12 | RFC4 | replication factor C subunit 4 [Source:HGNC Symbol;Acc:HGNC:9972] | 897 | 0.543 | 0.0231 | No |
| 13 | NSF | "N-ethylmaleimide sensitive factor, vesicle fusing ATPase [Source:HGNC Symbol;Acc:HGNC:8016]" | 908 | 0.540 | 0.0286 | No |
| 14 | DDX23 | DEAD-box helicase 23 [Source:HGNC Symbol;Acc:HGNC:17347] | 913 | 0.538 | 0.0345 | No |
| 15 | PMS2P1 | "PMS1 homolog 2, mismatch repair system component pseudogene 1 [Source:HGNC Symbol;Acc:HGNC:9123]" | 917 | 0.537 | 0.0406 | No |
| 16 | RFC1 | replication factor C subunit 1 [Source:HGNC Symbol;Acc:HGNC:9969] | 931 | 0.531 | 0.0457 | No |
| 17 | DHX29 | DExH-box helicase 29 [Source:HGNC Symbol;Acc:HGNC:15815] | 1012 | 0.515 | 0.0454 | No |
| 18 | MCM6 | minichromosome maintenance complex component 6 [Source:HGNC Symbol;Acc:HGNC:6949] | 1131 | 0.490 | 0.0418 | No |
| 19 | HSPA1L | heat shock protein family A (Hsp70) member 1 like [Source:HGNC Symbol;Acc:HGNC:5234] | 1139 | 0.488 | 0.0469 | No |
| 20 | GET3 | "guided entry of tail-anchored proteins factor 3, ATPase [Source:HGNC Symbol;Acc:HGNC:752]" | 1161 | 0.485 | 0.0509 | No |
| 21 | SMC2 | structural maintenance of chromosomes 2 [Source:HGNC Symbol;Acc:HGNC:14011] | 1190 | 0.479 | 0.0543 | No |
| 22 | RFC2 | replication factor C subunit 2 [Source:HGNC Symbol;Acc:HGNC:9970] | 1247 | 0.467 | 0.0553 | No |
| 23 | ATP6V1H | ATPase H+ transporting V1 subunit H [Source:HGNC Symbol;Acc:HGNC:18303] | 1304 | 0.457 | 0.0562 | No |
| 24 | CHD1L | chromodomain helicase DNA binding protein 1 like [Source:HGNC Symbol;Acc:HGNC:1916] | 1320 | 0.454 | 0.0603 | No |
| 25 | DDX60 | DExD/H-box helicase 60 [Source:HGNC Symbol;Acc:HGNC:25942] | 1385 | 0.445 | 0.0604 | No |
| 26 | PMS2P3 | "PMS1 homolog 2, mismatch repair system component pseudogene 3 [Source:HGNC Symbol;Acc:HGNC:9128]" | 1412 | 0.442 | 0.0635 | No |
| 27 | HSPA4L | heat shock protein family A (Hsp70) member 4 like [Source:HGNC Symbol;Acc:HGNC:17041] | 1485 | 0.431 | 0.0628 | No |
| 28 | SPAST | spastin [Source:HGNC Symbol;Acc:HGNC:11233] | 1502 | 0.429 | 0.0665 | No |
| 29 | DDX6 | DEAD-box helicase 6 [Source:HGNC Symbol;Acc:HGNC:2747] | 1687 | 0.401 | 0.0566 | No |
| 30 | OLA1 | Obg like ATPase 1 [Source:HGNC Symbol;Acc:HGNC:28833] | 1791 | 0.385 | 0.0530 | No |
| 31 | PSMC5 | "proteasome 26S subunit, ATPase 5 [Source:HGNC Symbol;Acc:HGNC:9552]" | 1987 | 0.362 | 0.0418 | No |
| 32 | CLU | clusterin [Source:HGNC Symbol;Acc:HGNC:2095] | 2083 | 0.352 | 0.0384 | No |
| 33 | MTREX | Mtr4 exosome RNA helicase [Source:HGNC Symbol;Acc:HGNC:18734] | 2138 | 0.346 | 0.0381 | No |
| 34 | KIF1B | kinesin family member 1B [Source:HGNC Symbol;Acc:HGNC:16636] | 2173 | 0.341 | 0.0394 | No |
| 35 | ATP5F1C | ATP synthase F1 subunit gamma [Source:HGNC Symbol;Acc:HGNC:833] | 2190 | 0.339 | 0.0421 | No |
| 36 | IFIH1 | interferon induced with helicase C domain 1 [Source:HGNC Symbol;Acc:HGNC:18873] | 2204 | 0.338 | 0.0450 | No |
| 37 | DHX57 | DExH-box helicase 57 [Source:HGNC Symbol;Acc:HGNC:20086] | 2262 | 0.331 | 0.0443 | No |
| 38 | CCT6B | chaperonin containing TCP1 subunit 6B [Source:HGNC Symbol;Acc:HGNC:1621] | 2333 | 0.323 | 0.0426 | No |
| 39 | TOR1B | torsin family 1 member B [Source:HGNC Symbol;Acc:HGNC:11995] | 2355 | 0.320 | 0.0446 | No |
| 40 | ORC1 | origin recognition complex subunit 1 [Source:HGNC Symbol;Acc:HGNC:8487] | 2525 | 0.300 | 0.0348 | No |
| 41 | NUBP1 | nucleotide binding protein 1 [Source:HGNC Symbol;Acc:HGNC:8041] | 2568 | 0.295 | 0.0349 | No |
| 42 | NUBPL | nucleotide binding protein like [Source:HGNC Symbol;Acc:HGNC:20278] | 2569 | 0.295 | 0.0383 | No |
| 43 | BBS10 | Bardet-Biedl syndrome 10 [Source:HGNC Symbol;Acc:HGNC:26291] | 2688 | 0.280 | 0.0322 | No |
| 44 | KIF20B | kinesin family member 20B [Source:HGNC Symbol;Acc:HGNC:7212] | 2714 | 0.278 | 0.0335 | No |
| 45 | ABCB6 | ATP binding cassette subfamily B member 6 (Langereis blood group) [Source:HGNC Symbol;Acc:HGNC:47] | 2717 | 0.278 | 0.0366 | No |
| 46 | RUVBL2 | RuvB like AAA ATPase 2 [Source:HGNC Symbol;Acc:HGNC:10475] | 2778 | 0.272 | 0.0350 | No |
| 47 | VPS4B | vacuolar protein sorting 4 homolog B [Source:HGNC Symbol;Acc:HGNC:10895] | 2783 | 0.271 | 0.0379 | No |
| 48 | DDX4 | DEAD-box helicase 4 [Source:HGNC Symbol;Acc:HGNC:18700] | 2788 | 0.271 | 0.0407 | No |
| 49 | KATNA1 | katanin catalytic subunit A1 [Source:HGNC Symbol;Acc:HGNC:6216] | 2891 | 0.261 | 0.0357 | No |
| 50 | ABCG5 | ATP binding cassette subfamily G member 5 [Source:HGNC Symbol;Acc:HGNC:13886] | 2942 | 0.256 | 0.0347 | No |
| 51 | ABCD2 | ATP binding cassette subfamily D member 2 [Source:HGNC Symbol;Acc:HGNC:66] | 3031 | 0.246 | 0.0306 | No |
| 52 | MYH8 | myosin heavy chain 8 [Source:HGNC Symbol;Acc:HGNC:7578] | 3119 | 0.238 | 0.0265 | No |
| 53 | CCT4 | chaperonin containing TCP1 subunit 4 [Source:HGNC Symbol;Acc:HGNC:1617] | 3120 | 0.238 | 0.0293 | No |
| 54 | DDX50 | DExD-box helicase 50 [Source:HGNC Symbol;Acc:HGNC:17906] | 3167 | 0.234 | 0.0284 | No |
| 55 | SMARCA2 | "SWI/SNF related, matrix associated, actin dependent regulator of chromatin, subfamily a, member 2 [Source:HGNC Symbol;Acc:HGNC:11098]" | 3281 | 0.222 | 0.0220 | No |
| 56 | DDX1 | DEAD-box helicase 1 [Source:HGNC Symbol;Acc:HGNC:2734] | 3305 | 0.221 | 0.0228 | No |
| 57 | CCT7 | chaperonin containing TCP1 subunit 7 [Source:HGNC Symbol;Acc:HGNC:1622] | 3464 | 0.206 | 0.0127 | No |
| 58 | HSPA14 | heat shock protein family A (Hsp70) member 14 [Source:HGNC Symbol;Acc:HGNC:29526] | 3506 | 0.202 | 0.0118 | No |
| 59 | CLPB | caseinolytic mitochondrial matrix peptidase chaperone subunit B [Source:HGNC Symbol;Acc:HGNC:30664] | 3514 | 0.201 | 0.0136 | No |
| 60 | VCP | valosin containing protein [Source:HGNC Symbol;Acc:HGNC:12666] | 3518 | 0.201 | 0.0157 | No |
| 61 | ERCC2 | "ERCC excision repair 2, TFIIH core complex helicase subunit [Source:HGNC Symbol;Acc:HGNC:3434]" | 3544 | 0.198 | 0.0160 | No |
| 62 | ABCA3 | ATP binding cassette subfamily A member 3 [Source:HGNC Symbol;Acc:HGNC:33] | 3549 | 0.198 | 0.0180 | No |
| 63 | SMC4 | structural maintenance of chromosomes 4 [Source:HGNC Symbol;Acc:HGNC:14013] | 3574 | 0.196 | 0.0184 | No |
| 64 | ATAD2 | ATPase family AAA domain containing 2 [Source:HGNC Symbol;Acc:HGNC:30123] | 3597 | 0.194 | 0.0189 | No |
| 65 | PEX1 | peroxisomal biogenesis factor 1 [Source:HGNC Symbol;Acc:HGNC:8850] | 3655 | 0.190 | 0.0166 | No |
| 66 | MCM3 | minichromosome maintenance complex component 3 [Source:HGNC Symbol;Acc:HGNC:6945] | 3675 | 0.188 | 0.0173 | No |
| 67 | PSMC3 | "proteasome 26S subunit, ATPase 3 [Source:HGNC Symbol;Acc:HGNC:9549]" | 3977 | 0.158 | -0.0047 | No |
| 68 | DNAH6 | dynein axonemal heavy chain 6 [Source:HGNC Symbol;Acc:HGNC:2951] | 4074 | 0.149 | -0.0106 | No |
| 69 | DHX58 | DExH-box helicase 58 [Source:HGNC Symbol;Acc:HGNC:29517] | 4130 | 0.143 | -0.0132 | No |
| 70 | ATP5F1A | ATP synthase F1 subunit alpha [Source:HGNC Symbol;Acc:HGNC:823] | 4137 | 0.143 | -0.0121 | No |
| 71 | MKKS | MKKS centrosomal shuttling protein [Source:HGNC Symbol;Acc:HGNC:7108] | 4182 | 0.140 | -0.0139 | No |
| 72 | TRIP13 | thyroid hormone receptor interactor 13 [Source:HGNC Symbol;Acc:HGNC:12307] | 4184 | 0.139 | -0.0124 | No |
| 73 | MLH3 | mutL homolog 3 [Source:HGNC Symbol;Acc:HGNC:7128] | 4335 | 0.125 | -0.0228 | No |
| 74 | KIF16B | kinesin family member 16B [Source:HGNC Symbol;Acc:HGNC:15869] | 4355 | 0.124 | -0.0228 | No |
| 75 | DHX40 | DEAH-box helicase 40 [Source:HGNC Symbol;Acc:HGNC:18018] | 4358 | 0.124 | -0.0215 | No |
| 76 | UPF1 | UPF1 RNA helicase and ATPase [Source:HGNC Symbol;Acc:HGNC:9962] | 4434 | 0.118 | -0.0261 | No |
| 77 | DDX19B | DEAD-box helicase 19B [Source:HGNC Symbol;Acc:HGNC:2742] | 4500 | 0.113 | -0.0299 | No |
| 78 | CHD9 | chromodomain helicase DNA binding protein 9 [Source:HGNC Symbol;Acc:HGNC:25701] | 4520 | 0.112 | -0.0301 | No |
| 79 | DDX28 | DEAD-box helicase 28 [Source:HGNC Symbol;Acc:HGNC:17330] | 4602 | 0.105 | -0.0353 | No |
| 80 | PMS2P5 | "PMS1 homolog 2, mismatch repair system component pseudogene 5 [Source:HGNC Symbol;Acc:HGNC:9130]" | 4623 | 0.103 | -0.0357 | No |
| 81 | TOR1A | torsin family 1 member A [Source:HGNC Symbol;Acc:HGNC:3098] | 4646 | 0.100 | -0.0363 | No |
| 82 | KIF5A | kinesin family member 5A [Source:HGNC Symbol;Acc:HGNC:6323] | 4661 | 0.099 | -0.0362 | No |
| 83 | C10orf88 | chromosome 10 open reading frame 88 [Source:HGNC Symbol;Acc:HGNC:25822] | 4677 | 0.098 | -0.0363 | No |
| 84 | ASCC3 | activating signal cointegrator 1 complex subunit 3 [Source:HGNC Symbol;Acc:HGNC:18697] | 4689 | 0.097 | -0.0360 | No |
| 85 | TOR3A | torsin family 3 member A [Source:HGNC Symbol;Acc:HGNC:11997] | 4690 | 0.097 | -0.0349 | No |
| 86 | MYH4 | myosin heavy chain 4 [Source:HGNC Symbol;Acc:HGNC:7574] | 4702 | 0.096 | -0.0346 | No |
| 87 | TRAP1 | TNF receptor associated protein 1 [Source:HGNC Symbol;Acc:HGNC:16264] | 4756 | 0.090 | -0.0378 | No |
| 88 | DDX47 | DEAD-box helicase 47 [Source:HGNC Symbol;Acc:HGNC:18682] | 4789 | 0.088 | -0.0393 | No |
| 89 | BLM | BLM RecQ like helicase [Source:HGNC Symbol;Acc:HGNC:1058] | 4843 | 0.084 | -0.0425 | No |
| 90 | SMC1A | structural maintenance of chromosomes 1A [Source:HGNC Symbol;Acc:HGNC:11111] | 4942 | 0.077 | -0.0493 | No |
| 91 | SPATA5L1 | spermatogenesis associated 5 like 1 [Source:HGNC Symbol;Acc:HGNC:28762] | 4982 | 0.074 | -0.0516 | No |
| 92 | KIFC1 | kinesin family member C1 [Source:HGNC Symbol;Acc:HGNC:6389] | 4997 | 0.073 | -0.0518 | No |
| 93 | DDX41 | DEAD-box helicase 41 [Source:HGNC Symbol;Acc:HGNC:18674] | 5004 | 0.073 | -0.0514 | No |
| 94 | ATAD5 | ATPase family AAA domain containing 5 [Source:HGNC Symbol;Acc:HGNC:25752] | 5020 | 0.071 | -0.0518 | No |
| 95 | KIF23 | kinesin family member 23 [Source:HGNC Symbol;Acc:HGNC:6392] | 5400 | 0.040 | -0.0813 | No |
| 96 | KIF13B | kinesin family member 13B [Source:HGNC Symbol;Acc:HGNC:14405] | 5448 | 0.037 | -0.0846 | No |
| 97 | YME1L1 | YME1 like 1 ATPase [Source:HGNC Symbol;Acc:HGNC:12843] | 5528 | 0.030 | -0.0905 | No |
| 98 | DHX8 | DEAH-box helicase 8 [Source:HGNC Symbol;Acc:HGNC:2749] | 5531 | 0.030 | -0.0903 | No |
| 99 | KIF14 | kinesin family member 14 [Source:HGNC Symbol;Acc:HGNC:19181] | 5565 | 0.027 | -0.0926 | No |
| 100 | KIF5C | kinesin family member 5C [Source:HGNC Symbol;Acc:HGNC:6325] | 5639 | 0.021 | -0.0981 | No |
| 101 | KIF3C | kinesin family member 3C [Source:HGNC Symbol;Acc:HGNC:6321] | 5696 | 0.016 | -0.1024 | No |
| 102 | DDX46 | DEAD-box helicase 46 [Source:HGNC Symbol;Acc:HGNC:18681] | 5744 | 0.012 | -0.1059 | No |
| 103 | BRIP1 | BRCA1 interacting helicase 1 [Source:HGNC Symbol;Acc:HGNC:20473] | 5885 | 0.003 | -0.1170 | No |
| 104 | PSMC6 | "proteasome 26S subunit, ATPase 6 [Source:HGNC Symbol;Acc:HGNC:9553]" | 5977 | -0.003 | -0.1241 | No |
| 105 | MCM4 | minichromosome maintenance complex component 4 [Source:HGNC Symbol;Acc:HGNC:6947] | 6003 | -0.005 | -0.1261 | No |
| 106 | KIF2C | kinesin family member 2C [Source:HGNC Symbol;Acc:HGNC:6393] | 6011 | -0.005 | -0.1266 | No |
| 107 | PEX6 | peroxisomal biogenesis factor 6 [Source:HGNC Symbol;Acc:HGNC:8859] | 6325 | -0.030 | -0.1510 | No |
| 108 | ATP13A2 | ATPase cation transporting 13A2 [Source:HGNC Symbol;Acc:HGNC:30213] | 6336 | -0.031 | -0.1514 | No |
| 109 | KIF18A | kinesin family member 18A [Source:HGNC Symbol;Acc:HGNC:29441] | 6414 | -0.036 | -0.1571 | No |
| 110 | KIF20A | kinesin family member 20A [Source:HGNC Symbol;Acc:HGNC:9787] | 6457 | -0.039 | -0.1599 | No |
| 111 | RAD50 | RAD50 double strand break repair protein [Source:HGNC Symbol;Acc:HGNC:9816] | 6474 | -0.040 | -0.1607 | No |
| 112 | DDX58 | DExD/H-box helicase 58 [Source:HGNC Symbol;Acc:HGNC:19102] | 6480 | -0.041 | -0.1606 | No |
| 113 | CCT8 | chaperonin containing TCP1 subunit 8 [Source:HGNC Symbol;Acc:HGNC:1623] | 6529 | -0.044 | -0.1639 | No |
| 114 | ABCG4 | ATP binding cassette subfamily G member 4 [Source:HGNC Symbol;Acc:HGNC:13884] | 6574 | -0.047 | -0.1668 | No |
| 115 | HYOU1 | hypoxia up-regulated 1 [Source:HGNC Symbol;Acc:HGNC:16931] | 6775 | -0.061 | -0.1820 | No |
| 116 | AK6 | adenylate kinase 6 [Source:HGNC Symbol;Acc:HGNC:49151] | 6824 | -0.065 | -0.1850 | No |
| 117 | KIF1A | kinesin family member 1A [Source:HGNC Symbol;Acc:HGNC:888] | 6829 | -0.065 | -0.1846 | No |
| 118 | ATAD2B | ATPase family AAA domain containing 2B [Source:HGNC Symbol;Acc:HGNC:29230] | 6896 | -0.072 | -0.1889 | No |
| 119 | KIF22 | kinesin family member 22 [Source:HGNC Symbol;Acc:HGNC:6391] | 6897 | -0.072 | -0.1881 | No |
| 120 | SMC3 | structural maintenance of chromosomes 3 [Source:HGNC Symbol;Acc:HGNC:2468] | 6950 | -0.076 | -0.1913 | No |
| 121 | PMS1 | "PMS1 homolog 1, mismatch repair system component [Source:HGNC Symbol;Acc:HGNC:9121]" | 6968 | -0.077 | -0.1918 | No |
| 122 | KIF2A | kinesin family member 2A [Source:HGNC Symbol;Acc:HGNC:6318] | 7155 | -0.092 | -0.2054 | No |
| 123 | SNRNP200 | small nuclear ribonucleoprotein U5 subunit 200 [Source:HGNC Symbol;Acc:HGNC:30859] | 7249 | -0.099 | -0.2116 | No |
| 124 | CCT3 | chaperonin containing TCP1 subunit 3 [Source:HGNC Symbol;Acc:HGNC:1616] | 7330 | -0.105 | -0.2167 | No |
| 125 | ATF7IP | activating transcription factor 7 interacting protein [Source:HGNC Symbol;Acc:HGNC:20092] | 7386 | -0.109 | -0.2198 | No |
| 126 | RSF1 | remodeling and spacing factor 1 [Source:HGNC Symbol;Acc:HGNC:18118] | 7393 | -0.110 | -0.2190 | No |
| 127 | DDX42 | DEAD-box helicase 42 [Source:HGNC Symbol;Acc:HGNC:18676] | 7447 | -0.114 | -0.2219 | No |
| 128 | RUVBL1 | RuvB like AAA ATPase 1 [Source:HGNC Symbol;Acc:HGNC:10474] | 7477 | -0.116 | -0.2228 | No |
| 129 | LONP1 | "lon peptidase 1, mitochondrial [Source:HGNC Symbol;Acc:HGNC:9479]" | 7531 | -0.121 | -0.2256 | No |
| 130 | ATRX | ATRX chromatin remodeler [Source:HGNC Symbol;Acc:HGNC:886] | 7551 | -0.123 | -0.2256 | No |
| 131 | BCS1L | "BCS1 homolog, ubiquinol-cytochrome c reductase complex chaperone [Source:HGNC Symbol;Acc:HGNC:1020]" | 7578 | -0.125 | -0.2262 | No |
| 132 | HSPA13 | heat shock protein family A (Hsp70) member 13 [Source:HGNC Symbol;Acc:HGNC:11375] | 7587 | -0.126 | -0.2254 | No |
| 133 | KIF18B | kinesin family member 18B [Source:HGNC Symbol;Acc:HGNC:27102] | 7636 | -0.129 | -0.2277 | No |
| 134 | EIF4A3 | eukaryotic translation initiation factor 4A3 [Source:HGNC Symbol;Acc:HGNC:18683] | 7646 | -0.130 | -0.2269 | No |
| 135 | AQR | aquarius intron-binding spliceosomal factor [Source:HGNC Symbol;Acc:HGNC:29513] | 7654 | -0.131 | -0.2259 | No |
| 136 | CCT5 | chaperonin containing TCP1 subunit 5 [Source:HGNC Symbol;Acc:HGNC:1618] | 7690 | -0.134 | -0.2271 | No |
| 137 | KIF26B | kinesin family member 26B [Source:HGNC Symbol;Acc:HGNC:25484] | 7770 | -0.139 | -0.2317 | No |
| 138 | RAD54L2 | RAD54 like 2 [Source:HGNC Symbol;Acc:HGNC:29123] | 7872 | -0.146 | -0.2380 | No |
| 139 | HSPA1A | heat shock protein family A (Hsp70) member 1A [Source:HGNC Symbol;Acc:HGNC:5232] | 7876 | -0.146 | -0.2366 | No |
| 140 | ERCC6 | "ERCC excision repair 6, chromatin remodeling factor [Source:HGNC Symbol;Acc:HGNC:3438]" | 7939 | -0.150 | -0.2397 | No |
| 141 | DDX49 | DEAD-box helicase 49 [Source:HGNC Symbol;Acc:HGNC:18684] | 7945 | -0.151 | -0.2384 | No |
| 142 | DDX18 | DEAD-box helicase 18 [Source:HGNC Symbol;Acc:HGNC:2741] | 7996 | -0.154 | -0.2405 | No |
| 143 | DHX30 | DExH-box helicase 30 [Source:HGNC Symbol;Acc:HGNC:16716] | 8111 | -0.164 | -0.2476 | No |
| 144 | PMS2 | "PMS1 homolog 2, mismatch repair system component [Source:HGNC Symbol;Acc:HGNC:9122]" | 8172 | -0.169 | -0.2504 | No |
| 145 | KIF17 | kinesin family member 17 [Source:HGNC Symbol;Acc:HGNC:19167] | 8183 | -0.170 | -0.2492 | No |
| 146 | PSMC1 | "proteasome 26S subunit, ATPase 1 [Source:HGNC Symbol;Acc:HGNC:9547]" | 8204 | -0.172 | -0.2488 | No |
| 147 | ATP1A2 | ATPase Na+/K+ transporting subunit alpha 2 [Source:HGNC Symbol;Acc:HGNC:800] | 8218 | -0.173 | -0.2478 | No |
| 148 | MYH3 | myosin heavy chain 3 [Source:HGNC Symbol;Acc:HGNC:7573] | 8258 | -0.176 | -0.2489 | No |
| 149 | IQCA1 | IQ motif containing with AAA domain 1 [Source:HGNC Symbol;Acc:HGNC:26195] | 8383 | -0.184 | -0.2565 | No |
| 150 | KIF21B | kinesin family member 21B [Source:HGNC Symbol;Acc:HGNC:29442] | 8456 | -0.190 | -0.2600 | No |
| 151 | HSPE1 | heat shock protein family E (Hsp10) member 1 [Source:HGNC Symbol;Acc:HGNC:5269] | 8487 | -0.193 | -0.2601 | No |
| 152 | CCT8L2 | chaperonin containing TCP1 subunit 8 like 2 [Source:HGNC Symbol;Acc:HGNC:15553] | 8635 | -0.203 | -0.2694 | No |
| 153 | ERCC3 | "ERCC excision repair 3, TFIIH core complex helicase subunit [Source:HGNC Symbol;Acc:HGNC:3435]" | 8669 | -0.206 | -0.2696 | No |
| 154 | MCM9 | minichromosome maintenance 9 homologous recombination repair factor [Source:HGNC Symbol;Acc:HGNC:21484] | 8781 | -0.215 | -0.2759 | No |
| 155 | DDX25 | DEAD-box helicase 25 [Source:HGNC Symbol;Acc:HGNC:18698] | 8815 | -0.218 | -0.2759 | No |
| 156 | ABCD3 | ATP binding cassette subfamily D member 3 [Source:HGNC Symbol;Acc:HGNC:67] | 8877 | -0.223 | -0.2782 | No |
| 157 | PSMC4 | "proteasome 26S subunit, ATPase 4 [Source:HGNC Symbol;Acc:HGNC:9551]" | 8886 | -0.224 | -0.2762 | No |
| 158 | CHD4 | chromodomain helicase DNA binding protein 4 [Source:HGNC Symbol;Acc:HGNC:1919] | 8917 | -0.226 | -0.2759 | No |
| 159 | ABCD1 | ATP binding cassette subfamily D member 1 [Source:HGNC Symbol;Acc:HGNC:61] | 8930 | -0.226 | -0.2743 | No |
| 160 | AFG3L2 | AFG3 like matrix AAA peptidase subunit 2 [Source:HGNC Symbol;Acc:HGNC:315] | 9045 | -0.235 | -0.2805 | No |
| 161 | HSP90B1 | heat shock protein 90 beta family member 1 [Source:HGNC Symbol;Acc:HGNC:12028] | 9134 | -0.242 | -0.2847 | No |
| 162 | HSPA8 | heat shock protein family A (Hsp70) member 8 [Source:HGNC Symbol;Acc:HGNC:5241] | 9140 | -0.243 | -0.2822 | No |
| 163 | HSPA9 | heat shock protein family A (Hsp70) member 9 [Source:HGNC Symbol;Acc:HGNC:5244] | 9173 | -0.246 | -0.2819 | No |
| 164 | EIF4A2 | eukaryotic translation initiation factor 4A2 [Source:HGNC Symbol;Acc:HGNC:3284] | 9251 | -0.252 | -0.2851 | No |
| 165 | ALPL | "alkaline phosphatase, biomineralization associated [Source:HGNC Symbol;Acc:HGNC:438]" | 9303 | -0.256 | -0.2861 | No |
| 166 | TCP1 | t-complex 1 [Source:HGNC Symbol;Acc:HGNC:11655] | 9431 | -0.267 | -0.2930 | No |
| 167 | RFC5 | replication factor C subunit 5 [Source:HGNC Symbol;Acc:HGNC:9973] | 9499 | -0.274 | -0.2951 | No |
| 168 | CLPX | caseinolytic mitochondrial matrix peptidase chaperone subunit X [Source:HGNC Symbol;Acc:HGNC:2088] | 9546 | -0.278 | -0.2955 | No |
| 169 | DDX24 | DEAD-box helicase 24 [Source:HGNC Symbol;Acc:HGNC:13266] | 9588 | -0.282 | -0.2955 | No |
| 170 | RHOBTB3 | Rho related BTB domain containing 3 [Source:HGNC Symbol;Acc:HGNC:18757] | 9783 | -0.300 | -0.3073 | Yes |
| 171 | CCT2 | chaperonin containing TCP1 subunit 2 [Source:HGNC Symbol;Acc:HGNC:1615] | 9789 | -0.300 | -0.3042 | Yes |
| 172 | HSPA4 | heat shock protein family A (Hsp70) member 4 [Source:HGNC Symbol;Acc:HGNC:5237] | 9797 | -0.301 | -0.3013 | Yes |
| 173 | YTHDC2 | YTH domain containing 2 [Source:HGNC Symbol;Acc:HGNC:24721] | 9872 | -0.309 | -0.3035 | Yes |
| 174 | GTF2F2 | general transcription factor IIF subunit 2 [Source:HGNC Symbol;Acc:HGNC:4653] | 9876 | -0.309 | -0.3002 | Yes |
| 175 | KIF1C | kinesin family member 1C [Source:HGNC Symbol;Acc:HGNC:6317] | 9942 | -0.316 | -0.3016 | Yes |
| 176 | SMARCA4 | "SWI/SNF related, matrix associated, actin dependent regulator of chromatin, subfamily a, member 4 [Source:HGNC Symbol;Acc:HGNC:11100]" | 10009 | -0.322 | -0.3031 | Yes |
| 177 | WRN | WRN RecQ like helicase [Source:HGNC Symbol;Acc:HGNC:12791] | 10010 | -0.323 | -0.2994 | Yes |
| 178 | MCM7 | minichromosome maintenance complex component 7 [Source:HGNC Symbol;Acc:HGNC:6950] | 10032 | -0.325 | -0.2972 | Yes |
| 179 | KIF15 | kinesin family member 15 [Source:HGNC Symbol;Acc:HGNC:17273] | 10058 | -0.327 | -0.2954 | Yes |
| 180 | HSP90AB1 | heat shock protein 90 alpha family class B member 1 [Source:HGNC Symbol;Acc:HGNC:5258] | 10060 | -0.327 | -0.2917 | Yes |
| 181 | RECQL | RecQ like helicase [Source:HGNC Symbol;Acc:HGNC:9948] | 10124 | -0.333 | -0.2928 | Yes |
| 182 | DHX32 | DEAH-box helicase 32 (putative) [Source:HGNC Symbol;Acc:HGNC:16717] | 10127 | -0.334 | -0.2890 | Yes |
| 183 | DDX51 | DEAD-box helicase 51 [Source:HGNC Symbol;Acc:HGNC:20082] | 10133 | -0.334 | -0.2855 | Yes |
| 184 | RECQL5 | RecQ like helicase 5 [Source:HGNC Symbol;Acc:HGNC:9950] | 10144 | -0.335 | -0.2824 | Yes |
| 185 | DHX35 | DEAH-box helicase 35 [Source:HGNC Symbol;Acc:HGNC:15861] | 10215 | -0.342 | -0.2840 | Yes |
| 186 | IGHMBP2 | immunoglobulin mu DNA binding protein 2 [Source:HGNC Symbol;Acc:HGNC:5542] | 10248 | -0.346 | -0.2825 | Yes |
| 187 | CHD3 | chromodomain helicase DNA binding protein 3 [Source:HGNC Symbol;Acc:HGNC:1918] | 10269 | -0.347 | -0.2800 | Yes |
| 188 | CHD2 | chromodomain helicase DNA binding protein 2 [Source:HGNC Symbol;Acc:HGNC:1917] | 10467 | -0.370 | -0.2913 | Yes |
| 189 | DHX15 | DEAH-box helicase 15 [Source:HGNC Symbol;Acc:HGNC:2738] | 10498 | -0.372 | -0.2893 | Yes |
| 190 | ABCA4 | ATP binding cassette subfamily A member 4 [Source:HGNC Symbol;Acc:HGNC:34] | 10554 | -0.379 | -0.2893 | Yes |
| 191 | HSPA6 | heat shock protein family A (Hsp70) member 6 [Source:HGNC Symbol;Acc:HGNC:5239] | 10561 | -0.379 | -0.2853 | Yes |
| 192 | DHX9 | DExH-box helicase 9 [Source:HGNC Symbol;Acc:HGNC:2750] | 10627 | -0.386 | -0.2860 | Yes |
| 193 | CCT6A | chaperonin containing TCP1 subunit 6A [Source:HGNC Symbol;Acc:HGNC:1620] | 10661 | -0.389 | -0.2841 | Yes |
| 194 | VPS4A | vacuolar protein sorting 4 homolog A [Source:HGNC Symbol;Acc:HGNC:13488] | 10768 | -0.401 | -0.2878 | Yes |
| 195 | MYO1E | myosin IE [Source:HGNC Symbol;Acc:HGNC:7599] | 10780 | -0.404 | -0.2839 | Yes |
| 196 | HSPA5 | heat shock protein family A (Hsp70) member 5 [Source:HGNC Symbol;Acc:HGNC:5238] | 10820 | -0.407 | -0.2823 | Yes |
| 197 | KIF3A | kinesin family member 3A [Source:HGNC Symbol;Acc:HGNC:6319] | 10848 | -0.410 | -0.2796 | Yes |
| 198 | WRNIP1 | WRN helicase interacting protein 1 [Source:HGNC Symbol;Acc:HGNC:20876] | 10909 | -0.418 | -0.2795 | Yes |
| 199 | CFTR | CF transmembrane conductance regulator [Source:HGNC Symbol;Acc:HGNC:1884] | 10920 | -0.420 | -0.2754 | Yes |
| 200 | MYO19 | myosin XIX [Source:HGNC Symbol;Acc:HGNC:26234] | 10938 | -0.423 | -0.2718 | Yes |
| 201 | DDX10 | DEAD-box helicase 10 [Source:HGNC Symbol;Acc:HGNC:2735] | 11029 | -0.434 | -0.2739 | Yes |
| 202 | G3BP1 | G3BP stress granule assembly factor 1 [Source:HGNC Symbol;Acc:HGNC:30292] | 11117 | -0.445 | -0.2756 | Yes |
| 203 | DDX21 | DExD-box helicase 21 [Source:HGNC Symbol;Acc:HGNC:2744] | 11120 | -0.446 | -0.2705 | Yes |
| 204 | NAV3 | neuron navigator 3 [Source:HGNC Symbol;Acc:HGNC:15998] | 11169 | -0.453 | -0.2691 | Yes |
| 205 | TDRD12 | tudor domain containing 12 [Source:HGNC Symbol;Acc:HGNC:25044] | 11283 | -0.469 | -0.2725 | Yes |
| 206 | DDX39A | DExD-box helicase 39A [Source:HGNC Symbol;Acc:HGNC:17821] | 11359 | -0.480 | -0.2729 | Yes |
| 207 | DDX56 | DEAD-box helicase 56 [Source:HGNC Symbol;Acc:HGNC:18193] | 11386 | -0.484 | -0.2693 | Yes |
| 208 | HSPD1 | heat shock protein family D (Hsp60) member 1 [Source:HGNC Symbol;Acc:HGNC:5261] | 11427 | -0.492 | -0.2667 | Yes |
| 209 | KIF25 | kinesin family member 25 [Source:HGNC Symbol;Acc:HGNC:6390] | 11464 | -0.497 | -0.2638 | Yes |
| 210 | BTAF1 | B-TFIID TATA-box binding protein associated factor 1 [Source:HGNC Symbol;Acc:HGNC:17307] | 11480 | -0.499 | -0.2592 | Yes |
| 211 | DDX52 | DExD-box helicase 52 [Source:HGNC Symbol;Acc:HGNC:20038] | 11484 | -0.499 | -0.2536 | Yes |
| 212 | MORC2 | MORC family CW-type zinc finger 2 [Source:HGNC Symbol;Acc:HGNC:23573] | 11523 | -0.504 | -0.2507 | Yes |
| 213 | NAV2 | neuron navigator 2 [Source:HGNC Symbol;Acc:HGNC:15997] | 11619 | -0.520 | -0.2522 | Yes |
| 214 | ATP2B1 | ATPase plasma membrane Ca2+ transporting 1 [Source:HGNC Symbol;Acc:HGNC:814] | 11648 | -0.524 | -0.2483 | Yes |
| 215 | ERCC6L | "ERCC excision repair 6 like, spindle assembly checkpoint helicase [Source:HGNC Symbol;Acc:HGNC:20794]" | 11663 | -0.527 | -0.2433 | Yes |
| 216 | LONP2 | "lon peptidase 2, peroxisomal [Source:HGNC Symbol;Acc:HGNC:20598]" | 11699 | -0.532 | -0.2398 | Yes |
| 217 | DDX17 | DEAD-box helicase 17 [Source:HGNC Symbol;Acc:HGNC:2740] | 11716 | -0.535 | -0.2348 | Yes |
| 218 | SMCHD1 | structural maintenance of chromosomes flexible hinge domain containing 1 [Source:HGNC Symbol;Acc:HGNC:29090] | 11732 | -0.538 | -0.2298 | Yes |
| 219 | COQ8B | coenzyme Q8B [Source:HGNC Symbol;Acc:HGNC:19041] | 11763 | -0.544 | -0.2258 | Yes |
| 220 | CHD5 | chromodomain helicase DNA binding protein 5 [Source:HGNC Symbol;Acc:HGNC:16816] | 11776 | -0.545 | -0.2204 | Yes |
| 221 | DDX19A | DEAD-box helicase 19A [Source:HGNC Symbol;Acc:HGNC:25628] | 11821 | -0.553 | -0.2174 | Yes |
| 222 | CHD8 | chromodomain helicase DNA binding protein 8 [Source:HGNC Symbol;Acc:HGNC:20153] | 11825 | -0.554 | -0.2112 | Yes |
| 223 | SMARCA5 | "SWI/SNF related, matrix associated, actin dependent regulator of chromatin, subfamily a, member 5 [Source:HGNC Symbol;Acc:HGNC:11101]" | 11838 | -0.557 | -0.2057 | Yes |
| 224 | DHX38 | DEAH-box helicase 38 [Source:HGNC Symbol;Acc:HGNC:17211] | 11860 | -0.562 | -0.2008 | Yes |
| 225 | ATAD3A | ATPase family AAA domain containing 3A [Source:HGNC Symbol;Acc:HGNC:25567] | 11920 | -0.577 | -0.1988 | Yes |
| 226 | RECQL4 | RecQ like helicase 4 [Source:HGNC Symbol;Acc:HGNC:9949] | 11927 | -0.578 | -0.1925 | Yes |
| 227 | RTEL1 | regulator of telomere elongation helicase 1 [Source:HGNC Symbol;Acc:HGNC:15888] | 11968 | -0.587 | -0.1888 | Yes |
| 228 | DDX54 | DEAD-box helicase 54 [Source:HGNC Symbol;Acc:HGNC:20084] | 11969 | -0.587 | -0.1820 | Yes |
| 229 | MDN1 | midasin AAA ATPase 1 [Source:HGNC Symbol;Acc:HGNC:18302] | 11973 | -0.588 | -0.1754 | Yes |
| 230 | HSPH1 | heat shock protein family H (Hsp110) member 1 [Source:HGNC Symbol;Acc:HGNC:16969] | 11995 | -0.592 | -0.1701 | Yes |
| 231 | SPG7 | "SPG7 matrix AAA peptidase subunit, paraplegin [Source:HGNC Symbol;Acc:HGNC:11237]" | 12020 | -0.597 | -0.1651 | Yes |
| 232 | DHX34 | DExH-box helicase 34 [Source:HGNC Symbol;Acc:HGNC:16719] | 12040 | -0.603 | -0.1596 | Yes |
| 233 | ACIN1 | apoptotic chromatin condensation inducer 1 [Source:HGNC Symbol;Acc:HGNC:17066] | 12059 | -0.609 | -0.1539 | Yes |
| 234 | TWNK | twinkle mtDNA helicase [Source:HGNC Symbol;Acc:HGNC:1160] | 12069 | -0.612 | -0.1475 | Yes |
| 235 | SUPV3L1 | Suv3 like RNA helicase [Source:HGNC Symbol;Acc:HGNC:11471] | 12087 | -0.616 | -0.1416 | Yes |
| 236 | EIF4A1 | eukaryotic translation initiation factor 4A1 [Source:HGNC Symbol;Acc:HGNC:3282] | 12111 | -0.623 | -0.1362 | Yes |
| 237 | KIF3B | kinesin family member 3B [Source:HGNC Symbol;Acc:HGNC:6320] | 12142 | -0.629 | -0.1312 | Yes |
| 238 | DNA2 | DNA replication helicase/nuclease 2 [Source:HGNC Symbol;Acc:HGNC:2939] | 12148 | -0.631 | -0.1243 | Yes |
| 239 | RAD54L | RAD54 like [Source:HGNC Symbol;Acc:HGNC:9826] | 12204 | -0.646 | -0.1211 | Yes |
| 240 | HSPA1B | heat shock protein family A (Hsp70) member 1B [Source:HGNC Symbol;Acc:HGNC:5233] | 12278 | -0.670 | -0.1191 | Yes |
| 241 | DDX31 | DEAD-box helicase 31 [Source:HGNC Symbol;Acc:HGNC:16715] | 12288 | -0.672 | -0.1120 | Yes |
| 242 | PIKFYVE | "phosphoinositide kinase, FYVE-type zinc finger containing [Source:HGNC Symbol;Acc:HGNC:23785]" | 12347 | -0.690 | -0.1085 | Yes |
| 243 | DDX27 | DEAD-box helicase 27 [Source:HGNC Symbol;Acc:HGNC:15837] | 12359 | -0.695 | -0.1013 | Yes |
| 244 | DHX16 | DEAH-box helicase 16 [Source:HGNC Symbol;Acc:HGNC:2739] | 12414 | -0.712 | -0.0973 | Yes |
| 245 | SRCAP | Snf2 related CREBBP activator protein [Source:HGNC Symbol;Acc:HGNC:16974] | 12542 | -0.769 | -0.0984 | Yes |
| 246 | CHD7 | chromodomain helicase DNA binding protein 7 [Source:HGNC Symbol;Acc:HGNC:20626] | 12591 | -0.791 | -0.0929 | Yes |
| 247 | DDX11 | DEAD/H-box helicase 11 [Source:HGNC Symbol;Acc:HGNC:2736] | 12607 | -0.803 | -0.0848 | Yes |
| 248 | KIF13A | kinesin family member 13A [Source:HGNC Symbol;Acc:HGNC:14566] | 12650 | -0.826 | -0.0785 | Yes |
| 249 | KIF5B | kinesin family member 5B [Source:HGNC Symbol;Acc:HGNC:6324] | 12662 | -0.835 | -0.0696 | Yes |
| 250 | DDX3Y | DEAD-box helicase 3 Y-linked [Source:HGNC Symbol;Acc:HGNC:2699] | 12728 | -0.890 | -0.0644 | Yes |
| 251 | HSP90AA1 | heat shock protein 90 alpha family class A member 1 [Source:HGNC Symbol;Acc:HGNC:5253] | 12730 | -0.893 | -0.0541 | Yes |
| 252 | CHD1 | chromodomain helicase DNA binding protein 1 [Source:HGNC Symbol;Acc:HGNC:1915] | 12745 | -0.908 | -0.0446 | Yes |
| 253 | DDX39B | DExD-box helicase 39B [Source:HGNC Symbol;Acc:HGNC:13917] | 12783 | -0.941 | -0.0366 | Yes |
| 254 | DDX3X | DEAD-box helicase 3 X-linked [Source:HGNC Symbol;Acc:HGNC:2745] | 12788 | -0.949 | -0.0258 | Yes |
| 255 | SMC5 | structural maintenance of chromosomes 5 [Source:HGNC Symbol;Acc:HGNC:20465] | 12791 | -0.952 | -0.0149 | Yes |
| 256 | DDX43 | DEAD-box helicase 43 [Source:HGNC Symbol;Acc:HGNC:18677] | 12800 | -0.969 | -0.0043 | Yes |
| 257 | DDX5 | DEAD-box helicase 5 [Source:HGNC Symbol;Acc:HGNC:2746] | 12834 | -1.038 | 0.0052 | Yes |
Table: GSEA details [plain text format]

  

Fig 2: GOMF\_ATP\_HYDROLYSIS\_ACTIVITY      
 Blue-Pink O' Gram in the Space of the Analyzed GeneSet

  

Fig 3: GOMF\_ATP\_HYDROLYSIS\_ACTIVITY: Random ES distribution      
 Gene set null distribution of ES for **GOMF\_ATP\_HYDROLYSIS\_ACTIVITY**
